# Ion Concentration Influences the Charge Transfer Due to a Water–Air Contact Line Moving over a Hydrophobic Surface: Charge Measurements and Theoretical Models

**DOI:** 10.1021/acs.langmuir.2c02716

**Published:** 2023-01-25

**Authors:** L. E. Helseth

**Affiliations:** Department of Physics and Technology, University of Bergen, Allegaten 55, 5020Bergen, Norway

## Abstract

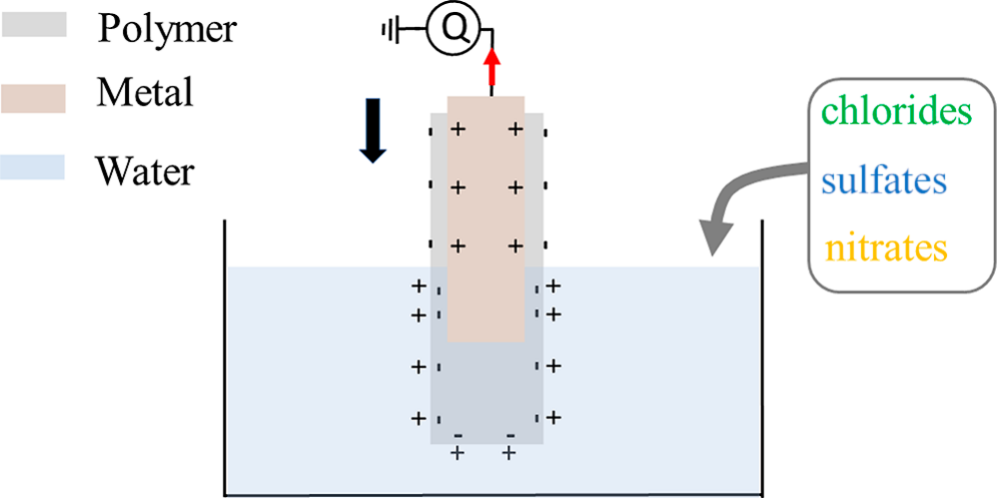

A metal electrode covered by an inert, hydrophobic polymer surface is dipped into water, and the charge transfer was measured as a function of ion concentration for different chlorides, sulfates, and nitrates. A generic behavior is observed wherein the charge transfer first increases and then decreases as the ion concentration increases. However, for acids, the charge transfer decreases monotonously with concentration and even reverses polarity. Two different models, both in which the charge transfer is attributed to removal of ions from the electrical double layer as the contact line passes by, are discussed and shown to provide possible explanations of the experimental data.

## 1 Introduction

The electrification of water coming in contact with an inert, hydrophobic solid surface has been investigated for more than a century.^1−5^ It is well known that a hydrophobic surface contacting pure water usually acquires a negative charge, while a corresponding positive charge remains in the liquid.^6−8^ It has been argued that hydroxide ions are involved in the formation of negative charges at the surface,^9,10^ while other studies have emphasized that the asymmetries and topological defects in the hydrogen bond network may be responsible.^11^ In general, the charge transfer depends on the solid surface roughness,^12^ the solid surface triboelectric state,^13−19^ and whether or not dissolution of ions takes place.^20,21^ In addition, the charge transfer is determined by the composition of the liquid,^22^ its flow rate,^23−27^ and the distance it moves over the solid surface.^28,29^ It is also known that the electrode structure^30^ and external electric fields have a significant impact on the amount and sign of the charge transfer.^31^

The influence of the ion concentration plays a role when liquid–solid interfaces charge up in the presence of flow.^32−35^ Measurements of the zeta potential at different salt concentrations demonstrate that for polytetrafluoroethylene (PTFE), the isoelectric point occurs for a pH about 3.^36,37^ X-ray photoelectron spectroscopy indicates that surface contaminations are unlikely to be the source of the proton release from the inert PTFE-particle surfaces, although the expected limit of detection is too uncertain to allow conclusive evidence thereof.^37^ Recent measurements using different types of measurements on different liquid motion systems appear to indicate that the charge transfer increases with the ion concentration for very dilute solutions but decreases with the ion concentration above 0.1–1 mM.^38−41^ However, at the same time, it is also known that some acids lead to a monotonic decay of charge transfer, and even reversal of charge, when the pH decreases.^27,39,42,43^ Some of these effects are well explained by an acid-base chemical equilibrium theory given in ref (43). For example, the theory of ref (43) explains very well why the charge transfer decreases with increasing concentration of acid, corresponding to a gradual decrease in pH, and the occurrence of an isoelectric point where the charge transfer is zero. Moreover, it also explains why one should expect an increase in charge transfer for increasing pH (up to pH = 10) or a decrease in charge transfer when a non-hydrolyzing salt is added. However, the theory does not naturally explain why the charge transfer first increases with the sodium chloride concentration until about 0.1–1 mM and then decreases for higher concentrations.^39,41^ The model proposed in ref (41) does explain this feature qualitatively, but it relies on assumptions that are difficult to validate. Furthermore, the experimental findings reported in refs (39)–^42^ are for a limited selection of salts dissolved in water, and it is of interest to find out what happens for a broader range of ions.

In the current work, these questions are addressed, and extensive measurements of the charge transfer for a range of different ions are undertaken on a robust and well-known hydrophobic fluoropolymer. Two different models based on ionic charge transfer are discussed and shown to be able to explain the experimental data.

## 2 Materials and Methods

### 2.1 Experimental Setup

The experimental setup used in this study was similar to that reported by the author in ref (41). A black, 2 mm thick polystyrene piece was cut to be 50 mm tall and 22 mm wide. A single-electrode device was made by attaching 0.03 mm thick aluminum tape to the polystyrene. The edge of the aluminum film was placed 15 mm from the lower edge of the polystyrene surface. An electrical wire was connected to the aluminum electrode. The aluminum electrode was covered entirely with fluorinated ethylene propylene (FEP) of thickness 50 μm (Dupont). A waterproof and non-leaching adhesive was used to seal the openings to avoid that water could come in contact with the metal electrode. The electrical resistance was measured before the experiments to ensure that no water leaked into the seal when the FEP was dipped into water. Upon dipping into water, the water could slide along the FEP-covered polystyrene for 15 mm before meeting the FEP-covered aluminum film. This length allowed reliable charging of the FEP surface near the position of the metal edge. FEP was used as a hydrophobic surface since it is known to provide a large contact electric response and is also not degraded in any known way by the chemical substances studied here. The FEP surface is hydrophobic for receding and advancing contact lines for the liquids under study, and the wetting properties, including advancing and receding contact angles, were reported in detail in refs (12)(41), and (44). The polymer–electrode system described earlier is hereafter called a single electrode as was also done in ref (41).

Deionized, ultrapure water (18.2 MΩ cm, Millipore) was used to make the solutions reported here. Only freshly created deionized water was used in order to avoid contamination due to dissolved gases (e.g., CO_2_) and ions from the container as much as possible. If one allowed the deionized water to rest in a plastic container in air for more than a day, a resistivity of the order of 1 MΩ cm was found, and the measurements of charge would also be altered. The single electrode was dipped into deionized water or a water containing ions. Solid powders or pellets of NaCl, KCl, Na_2_SO_4_ (anhydrous), CuSO_4_ (anhydrous), ZnSO_4_·7H_2_0, KNO_3_, NaNO_3_, and ZnNO_3_·6H_2_0 were purchased from Sigma-Aldrich. MnCl_2_ was obtained from Alfa Aesar. The solids were dissolved in deionized water to make solutions of different concentrations. Readymade solutions of 1 M HCl, 1 M HNO_3_, 1 M H_2_SO_4_, and 1 M HCOOH were purchased from Sigma-Aldrich.

A polystyrene beaker was filled with an aqueous solution to a fixed liquid level of 70 mL. The FEP surface was hydrophobic for both advancing and receding contact lines as the device was dipped into and pulled out of water. None of the aqueous solutions used in this study were found to alter the wettability significantly compared to the values reported in refs (12)(41), and (44).

**Figure 1:**
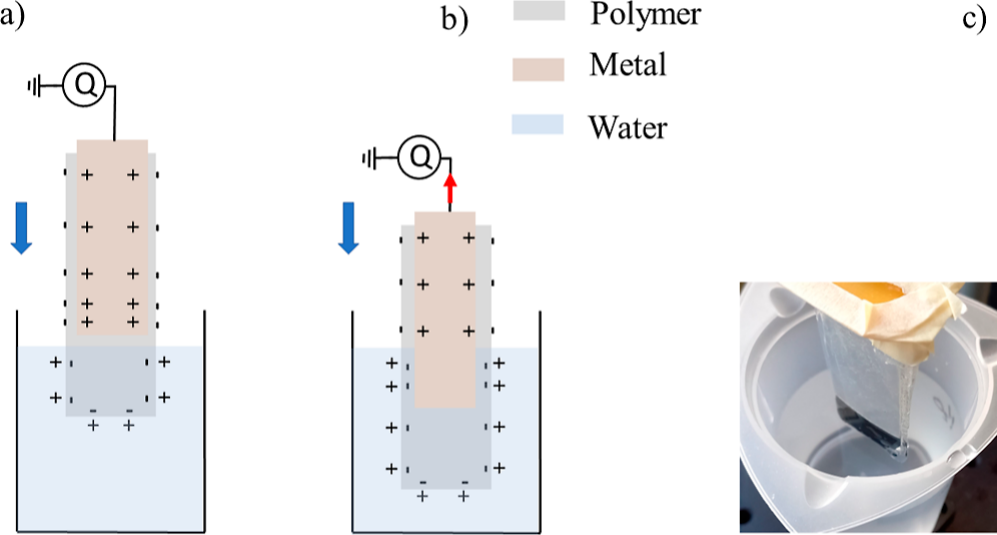
Simplified schematic drawing of the electrification as the single-electrode device is gradually moved into water above (a) and below (b) the electrode edge. A significant current (red arrow) is only observed as the three-phase contact line crosses the horizontal position of the metal edge, where there is a larger charge density as shown. (c) Single-electrode device positioned as in (a).

The single electrode made of a metal covered by a fluoropolymer was mounted on a cantilever and dipped into an aqueous solution using an electromagnetic shaker (Smart Materials GmbH) as described in refs (44) and (45). A schematic drawing of the dipping process is shown in Figure 1a,b. The charge transferred upon dipping the single electrode into an aqueous solution was measured using a Keithley 6514 electrometer, which measures the charge using charge amplifiers with high-input impedance. The electrometer is denoted “*Q*” in Figure 1a.

Both the amplitude and frequency of the electromagnetic shaker were controlled using a signal generator, and the oscillation amplitude and frequency of the single electrode were monitored using an ultrasonic probe in the same manner as reported in ref (45). The main charge transfer occurs when the region of the FEP located near the position of the metal edge was dipped into or retracted from the liquid since there is a larger concentration of charge here as shown in Figure 1 and explained in ref (41). The electrometer, therefore, measures the charge transfer in this region near the metal electrode (typically of the order of 1 mm), and its extension is discussed in more detail in Section 3.3.

In order to have repeatable measurements of the charge, it was decided that the easiest way to do this in a controllable manner was to let the single electrode be mounted on a vibrating cantilever, also done in ref (41). Using too small cantilever vibration amplitudes would fail to transfer all the charge near the electrode edge due to insufficient translational motion. The frequency range could be tuned somewhat without considerable consequences, but too fast oscillations lead to splashing and instabilities. While the vibration system used here does not work at velocities smaller than about 0.05 m/s, manual experiments pushing down the cantilever manually suggest that complete charge transfer may occur when the single electrode is moved slowly (0.01 m/s) or relatively quickly (0.1 m/s) over a sufficient long distance. This suggests that one upon moving the contact line provides a much bigger shear force than the minimum required to remove the charge. To obtain reliable and repeatable measurements, it was found that an oscillation amplitude of 8 mm at a frequency of 2.3 Hz, corresponding to a velocity of about 0.1 m/s, gave complete and repeatable charge transfer and stable operation over many hours and repeatability when the experiment was repeated several months after each other. These settings were used for all the experiments reported here.

### 2.2 Charge Measurements

**Figure 2:**
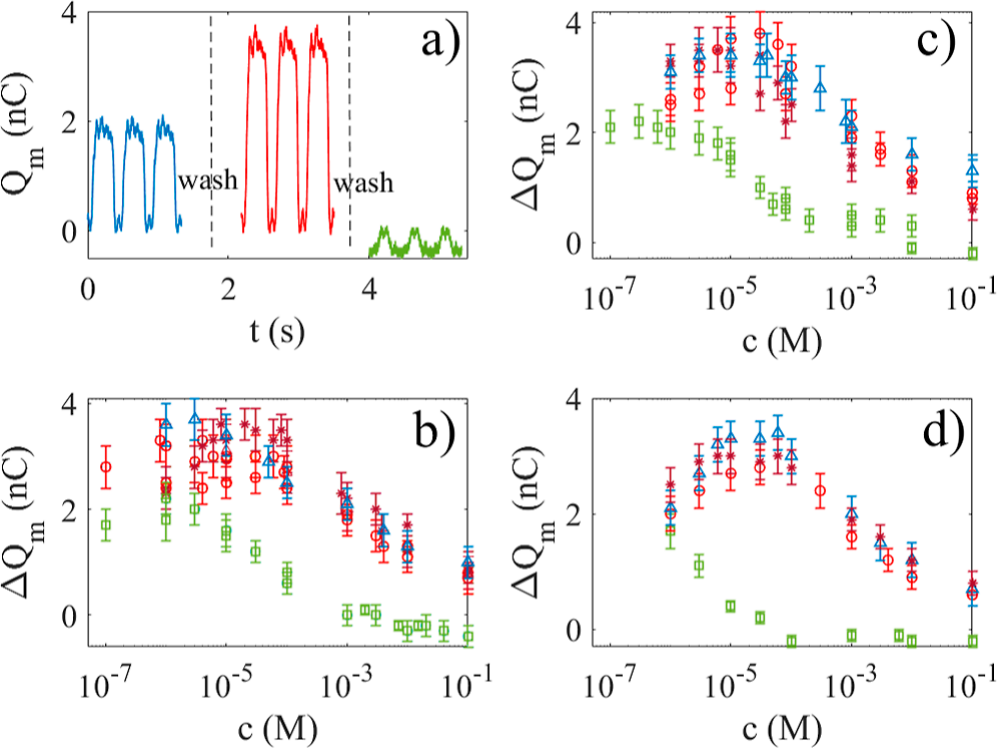
(a) Measured charge vs time when the polymer–electrode system is dipped into deionized water (blue), 0.08 mM NaCl (red), and 10 mM HCl (green). For every experiment, there is a washing step in between as described in the text, represented by the dashed vertical lines. In (b), the measured change in charge is shown when the polymer is dipped in a solution of HCl (green squares), KCl (red circles), NaCl (brown stars), and MnCl_2_ (blue triangles). In (c), the green squares correspond to H_2_SO_4_, the red circles to Na_2_SO_4_, the brown stars to CuSO_4_, and the blue triangles to ZnSO_4_. In (d), the green squares correspond to HNO_3_, the red circles to KNO_3_, the brown stars to ZnNO_3_, and the blue triangles to NaNO_3_.

The electrometer measured the charge *Q*_m_ as a function of time as the single electrode is dipped into the aqueous solution. An example of dipping the single electrode into 70 mL of deionized water is shown as a blue line in Figure 2a. It is seen that the maximum charge is about +2 nC when the single electrode is dipped into water and 0 nC when it is out of water. The charge transfer Δ*Q*_m_ is, therefore, 2 nC. The red line in Figure 2a shows the transferred charge when the single electrode is dipped into 0.08 mM NaCl. It is seen that the maximum charge transfer measured has increased to nearly +4 nC. On the other hand, if the single electrode is dipped into 10 mM HCl (green line), the charge transferred is about −0.5 nC.

After an experimental run including ionic solutions, the single electrode was cleaned by dipping it in deionized water at least five times, then removing the water, and repeating this procedure three to four times until it was observed that charge transfer was back to about +2 nC as reported in Figure 2a.

In general, it was observed that the transferred charge came back to the original +2.0 nC to within ±0.2 nC following this procedure. Lower values of about +1.5 nC were observed if the single electrode had been lying unused in sunlight for several weeks. Under such circumstances, it was necessary to activate the FEP surface by prolonged dipping into ionic solutions followed by cleaning in deionized water before use. After wiping with methanol and activation by numerous dipping in water, the charge transfer was once more 2.0 ± 0.2 nC in deionized water. The charge transfer was also found to be lower if deionized water that had been exposed to air in a plastic or glass container for more than a day was used. This reduction in charge transfer is most likely due to uptake of CO_2_, causing an increase in carbonic acid. Increasing the concentration of bulk hydrogen ions then causes a reduced charge transfer. This was prevented by using fresh deionized water, typically used within 1–2 h after tapping. Under such circumstances, the obtained charge was within the limits 2.0 ± 0.2 nC.

Although the FEP polymer surface used here is known to be inert toward most chemicals, it is known to react with strong reductive agents such as alkali metals or photochemically activated sodium sulfite.^46^ In the current study, no such chemicals or exposures are made on purpose, although the electrodes were allowed to remain in normal sunlight for a few weeks between some of the measurements. However, as mentioned earlier, it was found that the surfaces did not degrade and could always be reactivated to provide repeatable results to within the uncertainty stated.

Curves similar to those in Figure 2a were measured for a range of different ionic solutions, and the charge transfer Δ*Q*_m_ was recorded as a function of ion concentration. Due to fluctuations and the measurement error of the instrument used, the uncertainty in each individual measurement is about ±0.1 nC. If recording oscillations like those of Figure 2a for a few minutes, one would observe slightly larger uncertainties in the average values, each of which are recorded as error bars in Figure 2b–d.

Figure 2b shows the measured charge transfer for a single-electrode dipped in different chlorides for concentrations between 0.1 μM and 0.1 M. Before each series, the Δ*Q*_m_ for deionized water was measured and found to be 2.0 ± 0.2 nC. The smallest concentrations were made by sequentially diluting samples that were initially 0.1 M. It is noted that KCl (red circles), NaCl (brown stars), and MnCl_2_ (blue triangles) show transferred charge larger than 2.0 nC even for the smallest concentrations used here (0.1 μM). Moreover, for these salt solutions, there appears to be a general trend with an increase in charge transfer until a concentration of about 10–100 μM, after which it decreases monotonously. This is in contrast to a solution of HCl (green squares), which for the smallest concentrations does not alter the charge transfer to within the experimental uncertainty, but beyond 5 μM gives rise to a strong decrease. Moreover, it is found that for HCl concentrations in the range 0.1–1 mM, the charge transfer changes sign and becomes negative, as was also reported in refs (39) and (43). This reversal of charge transfer can also be seen from the green curve in Figure 2a.

The trends observed for sulfates in Figure 2c and nitrates in Figure 2d exhibit the same qualitative behavior as observed in Figure 2b for chlorides. The sulfates Na_2_SO_4_, CuSO_4_, and ZnSO_4_ and the nitrates KNO_3_, ZnNO_3_, and NaNO_3_ result in a charge transfer that grows from 2 nC (for pure water) to little less than 4 nC at 10–100 μM, after which the charge transfer starts to decay monotonously to about 1 nC at 0.1 M. While there are small individual variations between the salts, they are smaller than the experimental uncertainties. One is, therefore, led to conclude that these salts all give rise to similar charge transfer behavior.

However, both H_2_SO_4_ and HNO_3_ give rise to a monotonously decrease in charge transfer for concentrations exceeding a few mM (millimolar) and eventually shift sign from positive to negative at higher concentrations. While it was observed that this change in sign occurred at 0.1 mM for HNO_3_, no such shift was observed for H_2_SO_4_ until concentrations of 10 mM. It should be mentioned that the charge reversal observed at this concentration of H_2_SO_4_ was not stable as sometimes positive and sometimes negative charge transfer could be recorded. Thus, there appears to be a small, but noticeable, difference in the behavior of the acids HCl, H_2_SO_4_, and HNO_3_, in particular, at larger concentrations. However, their overall behavior appears to be the same.

## 3 Results and Discussion

### 3.1 Preliminary Estimates

Some simple estimates are helpful when trying to grasp how fast and how much charge builds up at the polymer surface. The water molecule in bulk water has a radius of about 0.1 nm, which means that in an area of about 10^–4^ m^2^ (1 cm^2^), there could be about 10^–4^ m^2^/(10^–10^ m)^2^ = 10^16^ water molecules close to the inert solid surface. If each water could polarize the surface with a charge −1.6 × 10^–19^ C, the total charge would be about −10^–3^ C. However, since the actual charge transfer is of the order of 1 nC/cm^2^, it appears that only a fraction of about 10^–6^ of the molecules contribute with an electronic charge during contact electrification.

Let us now assume that we are interested in finding out how large the concentration of additional ions would need to be to make a contribution to the contact electrification. Considerable experimental evidence points to the fact that contact electrification (i.e., the charge buildup) in single electrodes (i.e., no direct contact with conductors) does not happen over a timescale smaller than 10^–3^ s for deionized water or small ion concentrations.^25,26,41^ Within this time, the ions should diffuse and drift toward the polymer surface over a length *L*. The diffusion timescale is τ_diff_ = *L*^2^/*D*, where *D* is the diffusion coefficient. The drift timescale is τ_drift_ = *L*/*v*, which upon relating the electric field *E* from the polymer surface and the mobility μ according to *v* = μ*E* gives τ_drift_ = *L*/μ*E*. Moreover, if the system is not driven too far from equilibrium, it is reasonable to assume that the Einstein relationship, μ = *qD*/*k*_B_*T*, is valid. Here, *k*_B_ is Boltzmann and *T* is the temperature. If one also estimates the electric field as the voltage *U* divided by the length *L*, *E* = *U*/*L*, the drift time can now be written as τ_drift_ = τ_diff_(*k*_B_*T*/*qU*). Within this estimate, it is seen that the drift time is smaller than the diffusion time if *qU* < *k*_B_*T*. It is probable that *qU* > *k*_B_*T* may occur as the ion approaches the polymer surface since the electric field may be large here, or if the ions are somehow confined in a surface structure where the hydration shell is partially removed. However, it is also reasonable to assume that for hydrated ions sufficiently far from the solid surface, these electric fields are screened during most of the charge’s movement toward the polymer surface such that the path is governed by diffusion wherein the diffusion time is not smaller than τ_diff_ ≈ 10^–3^ s. If one also assumes that the bulk water molecules diffuse with the diffusion constant *D* = 10^–9^ m^2^/s toward the surface, the water molecules or ions will be able to diffuse roughly a distance . With a surface area of 10^–4^ m^2^, the volume from which ions can be taken is 10^–10^ m^3^ = 10^–7^ L. If one requires that the additional ions also contribute with 10^–9^ C in the given area 10^–4^ m^2^ and that the contributed charge is 96 485 C/mol (Faraday’s constant), there must be approximately 10^–9^ C/(10^5^ C/mol) = 10^–14^ mol of ions contributing in the volume. The concentration of ions available in the volume needed to generate the additional 10^–9^ C on the 1 cm^2^ polymer surface is, therefore, 10^–14^ mol/10^–7^*L* = 10^–7^ M. Comparing with the experimental data of Figure 2, it is clear that the concentrations larger than 10^–7^ M are needed to contribute additional 10^–9^ C. Thus, if the numbers used in the estimate are correct, there are enough diffused ions in the vicinity of the polymer surface to explain the buildup of charge observed. Adding drift would only allow an even larger number of ions participating, thus potentially allowing larger charge transfer.

According to the above simple estimates, the ions may diffuse toward the surface over a length scale of about 1000 nm to provide the required charge. In ref (39), it was hypothesized that only ions within about 20 nm from the surface could contribute to contact electrification, and estimates based on this suggested that ions cannot explain the increase in charge observed when ions are added to the solution. This is clearly at odds with the above estimate, and further experimental studies are, therefore, needed to resolve the nanoscale ion diffusion and drift near the interface to find out which estimates are correct, but this is outside the scope of the current work. However, we will in the current work demonstrate that ions can indeed explain the additional charge transfer observed in this and other studies relating to aqueous media.

### 3.2 Chemical Equilibrium Theory

Based on the observations seen in Figure 2, it appears that the behavior of the charge transfer is qualitatively, and to a large degree quantitatively, the same for all other ions than the hydrogen ions.

With this in mind, a logical approach to model the system is through an acid-base chemical equilibrium theory as discussed in ref (43). The theory of ref (43) explains very well why the charge transfer decreases with increasing concentration of acid, corresponding to a gradual decrease in pH, and the occurrence of an isoelectric point where the charge transfer changes sign. It also explains the observed increase in charge transfer for increasing pH up to 10, as well as the decrease in charge transfer with salt concentration. However, the theory presented in ref (43) does not naturally explain why dissolved salts give rise to the same increase in charge transfer for small concentrations as is observed in Figure 2. In the current work, it is shown that by some modifications of the theory of ref (43), clarifying the various contributions to charge transfer, one may construct a chemical equilibrium theory which explains all the features mentioned earlier.

Consider a polymer surface dipped into water containing cations A^+^ and anions B^–^. Let us first assume that the water molecules interact with particular sites on the polymer surface denoted *P*_s_^p^ and form negatively charged species  on the surface. There are *N*_p_ sites denoted *P*_s_^p^. The surface-active protons denoted  are released near the polymer surface. This reaction can be written as
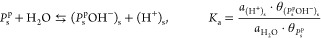
1Here, *K*_a_ is a dimensionless equilibrium constant following the description in ref (43),  is the water activity,  is the (dimensionless) fraction of polymer sites which here interact with water molecules, and  is the fraction of sites which initially take up negative charge as shown in eq 1. The surface activity of hydrogen ions  could be related to the excess bulk activity .^47,48^ Asymmetries and topological defects in the hydrogen bond network are expected to occur based on recent models,^11^ and these may lead to an energy  that depends on the bulk activity. Writing the surface activity as , one has for small bulk activities , where Δ*E*_0_ could be interpreted as the bulk activity-independent energy barrier and h is a constant which in most situations is negative since a bulk hydrogen ion concentration lowers the energy barrier for additional hydrogen ions to access the hydrophobic surface. For small bulk activities, this gives , which can be written as
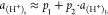
2where  and  are constants. If eq 2 is valid, one would observe that sufficiently low excess bulk hydrogen ion concentrations do not influence the surface hydrogen ion activity at the polymer sites described earlier.

The released surface-active hydrogen ions in eq 1 may form an electrical double layer with the negative charged species on the surface. We here argue that this reaction is different from that of eq 1 in that it is an aggregate formation of the electrical double layer which formulated in reaction form can be described as
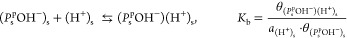
3Here,  denotes the loosely bound charges in the electrical double layer, forming a neutral entity, with surface fraction .  from eq 1 may move into a fluctuating hydrogen bond network with topological defects and asymmetries which contribute to  in eq 3. Thus, it should be noted that eqs 1 and 3 are not the same and that  is not the same as adsorbed water. The association of the hydrogenic charges with the electrical double layer is determined by the equilibrium constant *K*_b_. These aggregates may release hydrogen ions into the bulk if exposed to shear stress from fluid flow. The shear force required should depend on the electrostatic attraction as well as the drag forces provided by the three-phase contact line passing by. There might be a minimum shear force required to remove the charge, but based on the observations reported in Section 2, it is believed that the experimentally applied shear force is much larger than this minimum and that its particular behavior is of no consequence here.

In the presence of cations, one also has another possible formation of an electrical double layer, which in analogy with eq 3 can be written as
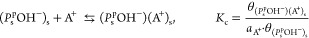
4Here,  is also loosely bound charges forming an electrical double layer with surface fraction . These aggregates may also release positively charged ions into the bulk when exposed to shear stress from fluid flow. A model assumption of eqs 3 and 4 is that both surface-active protons, , and the externally introduced cations, A^+^, occupy the negatively charged sites  induced by the interaction between the water molecules and the polymer surface.

In the model presented here, there are three species competing to occupy the sites on the polymer surface denoted *P*_s_^p^, resulting in either negatively charged or neutral sites. In total, the relevant occupancy should be the one as given in the following equation
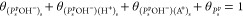
5

Combining eqs 1–5 gives the fraction of negative charges at the water–polymer interface in the presence of water.
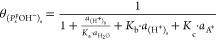
6

We will in the following assume that the activity of the cations is equal to the concentration such that *a*_A_^+^ = *c*. A fraction φ_–_ of the sites will release positive charge,  and , into the bulk if the three-phase contact line moves past them. This fraction can be expressed as
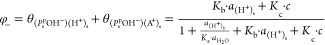
7When the contact line moves past these sites, positive charges are released into the bulk with a fraction as described according to eq 7. The increase in negative charge on the polymer surface when the hydrophobic polymer surface moves out of water is proportional to the fraction φ_–_. It should be emphasized that it is this fraction which will contribute to a change in electrostatic induction in the metal electrode attached to the polymer surface and therefore contribute to the measured charge. If there are *N*_p_ sites of type *P*_s_^p^, one may expect that the *N*_p_φ_–_ number of ions is revealed when the water front moves past the polymer surface.

So far, we have not accounted for any quenching as the concentration of cations increases. In ref (43), it was assumed that the activity of water molecules at the interface is quenched by added salt. The quenching is described by a factor γ_p_ = *a*_f_/(*a*_f_ + *a*_b_), where *a*_f_ is the activity of free water and *a*_b_ is the activity of water bound by ions. If one assumes that the sum of free and bound water molecules is constant and that the reaction between salt and water to create bound water is associated with an equilibrium constant *K*_qp_, then γ_p_ = 1/(1 + *K*_qp_*c*), where *c* is the concentration.^43^ This equation for γ_p_ must be included to account for the effective number of ions participating in the charge transfer and will be used in the modeling. The effective number of sites of type *P*_s_^p^ contributing to charge transfer is γ_p_N_p_, and the  number of ions is revealed when the water front moves past the polymer surface.

The special behavior induced by the acids displayed in Figure 2 warrants the introduction of a hydrogen ion-specific term to the charge transfer. In addition to the sites denoted *P*_s_^p^ which preferentially form negative sites, the hydrophobic polymer may also have sites *P*_s_^n^ of fraction , which tend to form positively charged surface species by associating with hydrogen ions from the bulk such that
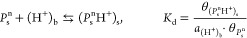
8where *K*_d_ is an equilibrium constant and  is the fraction of sites which initially take up positive charge as shown in eq 8. Unlike the preferential negatively charged sites denoted *P*_s_^p^, it is assumed here that the hydrogen ions from the bulk interact directly with the sites *P*_s_^n^ such that water molecules only act as catalyzers and do not participate in the reaction. Simulations support the idea that hydrated hydrogen and hydroxide ions behave differently in bulk,^49^ but less is known about their surface activity. Hydrated protons move in water through interconversion between relatively few hydrated complexes, which may aid a direct interaction with the polymer sites. There must be an asymmetry at the interface for hydrogen and hydroxide ions. Hydrogen ions are small with high mobility and can easily move through the asymmetries and defects in the hydrogen bond network near the surface without strongly interacting with water molecules. On the other hand, the larger and less mobile hydroxide ions must interact strongly with water.

It will be assumed that when the aqueous solution is removed, the weakly surface-bound hydrogen ions follow the liquid such that the positive charge associated with  is also removed. Thus, the change in positive surface charge upon shear flow is due to the sites  and not so much related to the anions B^–^. The data in Figure 2 suggest that major changes occur when one changes from hydrogen ions to any other cation, but similar major changes are not seen for any of the anions. For example, no major changes in the charge transfer behavior are seen when one changes from chloride ions to hydroxide ions, thus suggesting that added bulk hydroxide ions do not play a special role. Experimentally, it is also observed that the solid surface charges negatively in pure water. Together with the asymmetry discussed earlier and that the anions are larger and less mobile than the hydroxide ions, it is reasonable to assume that these anions do not play a particular role in the charge transfer. This may not be surprising since anions typically do not coordinate water molecules as efficiently as cations do. The occupancy of the sites on the polymer surface denoted *P*_s_^n^ must fulfill
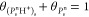
10As the waterfront is removed, there is an additional positive charge which is proportional to  on the dry polymer surface. Combining eqs 8–10 results in the fraction of sites contributing to a positive charge
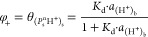
11

We assume that a fraction of charged sites that are uncovered when the three-phase contact line moves past the polymer surface, thus inducing an opposite electric charge in the metal electrode. There are *N*_n_ sites of type *P*_s_^n^. At low concentrations, quenching of water activity is not expected since the hydrogen ions are highly mobile in the hydrogen bond network. It is possible that the bulk hydrogen ion activity is quenched at higher bulk concentrations with a quenching of the type γ_n_ = 1/(1 + *K*_qn_*a*_H_^+^), where *K*_qn_ is an equilibrium constant. However, since the introduction of such a quenching factor γ_n_ does not improve the fit of the model presented in this section to the experimental data, it will not be considered in this section. The effective number of sites of type *P*_s_^p^ contributing to charge transfer is, therefore, *N*_n_, and the *N*_n_φ_+_ number of ions is revealed when the water front moves past the polymer surface.

If one assumes that the chemical reactions and physical interactions at the sites *P*_s_^p^ and *P*_s_^n^ are uncorrelated, the change in polymer surface charge as the polymer surface is moved out of water is
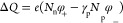
12where *e* = 1.602 × 10^–19^ C is the electronic charge. The entire change in charge results in a corresponding, but opposite, change in charge on the metal electrode which is measured by the electrometer. Therefore, the measured charge is Δ*Q*_m_ = −Δ*Q*. Assuming that the activities equal the concentrations, , , and using eqs 7 and 11 and γ_p_ = 1/(1 + *K*_qp_*c*) from ref (43) give
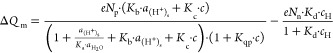
13

Two special cases of eq 13 are investigated in this work in an attempt to understand the experimental results of Figure 2. First, let us assume that the added bulk hydrogen ion concentration is zero, such that . The surface activity of hydrogen ions may still be nonzero due to the interaction between water and polymer sites, and the measured charge is
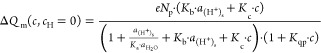
14

Thus, under such circumstances, the measured charge is always positive upon withdrawal from water, in agreement with experimental observations.

**Figure 3:**
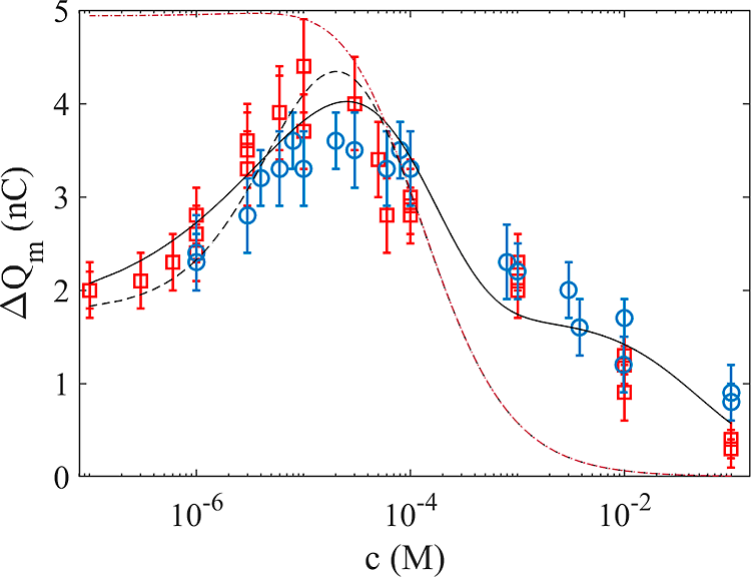
Measured contact charge transfer vs concentration of NaOH (red squares) and NaCl (blue circles). The dashed (black) line is a fit of eq 14 to the data with *K*_c_ = 2.0 × 10^5^ M^–1^, *K*_qp_ = 1.0 × 10^4^ M^–1^, *N*_p_ = 4.0 × 10^10^, and , whereas the solid (black) line is a fit of eq 17 to the data with *Q*_0p_ = 1.7 × 10^–9^ C, *AB*_p_ = −5.4 × 10^–7^ V m^2^, *K*_qp_ = 20 M^–1^, and *x*_sp_ = 60 × 10^–9^ m. The dashed–dotted (brown) line is a plot of eq 14 assuming a hypothetical case with  as described in the text.

The black dashed line in Figure 3 shows a fit of eq 14 to the experimental data with *K*_c_ = 2.0 × 10^5^ M^–1^, *K*_qp_ = 1.0 × 10^4^ M^–1^, *N*_p_ = 4.0 × 10^10^, and . It is assumed that  since giving this constant a finite value comparable to or larger than 1 does not increase the quality of the fitted curve, and one cannot determine it with confidence. This may, therefore, suggest that the equilibrium in eq 1 is driven entirely to the right when  as if most of the available sites *N*_p_ are occupied by charged species.

The chemical equilibrium model presented in eq 14 states that in the absence of additional ions, one has , and therefore, the charge transfer in pure deionized water is explained as a result of the product of surface proton activity and the equilibrium constant stating that how easy it is for the surface protons to participate in the electrical double layer. Increasing the number of sites available at the hydrophobic surface beyond *N*_p_ = 4.0 × 10^10^ would also increase the charge transfer. If 10^16^ water molecules are located in the innermost layer near the polymer surface, one estimates using *N*_p_ = 4.0 × 10^10^ that a fraction of 4 × 10^10^/10^16^ = 4 × 10^–6^ of these participate in the charge transfer, in reasonable agreement with the simple estimates presented in Section 3.1.

It should be emphasized that the equilibrium constants obtained from fitting eq 14 to the experimental data relate to the three-phase contact line moving past a hydrophobic surface and may, therefore, be different from those one may obtain using streaming currents in the presence of a continuous water front.^10,47^ One should also be careful when comparing the equilibrium constants in ref (43) with those introduced here since they have different meaning due to the differences in the theory presented here and the acid–base theory of ref (43). For example, the value *K*_c_ = 2.0 × 10^5^ M^–1^ obtained by fitting eq 14 to the experimental data in Figure 3 is much larger than the equilibrium constant *K*_Na_ = 2.0 × 10^–11^ M^–1^ reported in ref (43). In the current work, hydroxide ions are assumed to be tightly associated with the polymer sites, but the free hydroxide ions do not play a particular role as they did in the theory presented in ref (43). Moreover, had *K*_c_ in the current work been very small, eq 14 would have predicted monotonous decay in the charge transfer with increasing concentration, contrary to the observations in Figures 2 and 3. Therefore, a large *K*_c_ as found from the fitted data in Figure 3 is necessary to explain the peak in charge transfer observed. In ref (43), the small equilibrium constant *K*_Na_ facilitating sodium ion adsorption was suggested to be associated with the structure-making ability of sodium ions, i.e., related to its ability to coordinate surrounding water molecules. In the current work leading to eq 14 and a large *K*_c_, we argue that the observed experimental data are the result of the added cations’ ability to strongly associate with the available polymer sites competing with the surface protons. Interpreted in such a manner, the experimental data do not suggest a significant difference in the different cations’ ability as structure makers or breakers at low concentrations. Stated in another way, any differences between the non-hydrogenic cations when it comes to coordination of water and the formation of hydration shells that also result in differences in the charge transfer cannot be confidently resolved with the experimental technique used here.

The product  is composed of two constants which cannot be extracted separately. As an example, one could make the crude approximation that the surface proton activity is  for small concentrations, thus obtaining  and , which suggests that the protons may contribute more than cations A^+^ to the charge transfer. Only if the surface proton activity near the hydrophobic surface is much more than an order of magnitude larger than that of the bulk, the relative contribution of the non-hydrogenic cations to  is expected to be larger than that of surface protons to , but again the fitting of eq 14 to the experimental data does not allow further precision in this matter since  cannot be found independently. Note also that by increasing the product of the surface hydrogen ion activity and equilibrium constant to , one obtains the brown dashed–dotted line in Figure 3, which does not exhibit a maximum in the charge transfer with ion concentration. Thus, alterations in the surface activity of hydrogen ions may play a crucial role for whether a peak charge transfer is observed.

When salt is added to the deionized water, the model represented by eq 14 states that the number of ions participating in the electrical double layer should increase according to eq 4. The fraction of positions in the electrical double layer should in principle fill up until saturation according to eq 7. However, the nonzero quenching constant *K*_qp_ makes sure that increasing the concentration of ions results in reduced water activity and therefore reduced possibility for ions to participate in the charge that is removed from the electrical double layer. The peak observed in Figure 3 is, therefore, a compromise between the increasing filling fraction of the electrical double layer at small concentrations and the reduced water activity at higher concentrations. Since the quenching mechanism proposed in ref (43) is also assumed in the current work, the equilibrium constant *K*_pq_ should in principle be comparable to the *K*_q_ found in figure 5 in ref (43). However, the constant *K*_qp_ = 1.0 × 10^4^ M^–1^ found from fitting eq 14 to the experimental data is typically between 2 and 3 orders of magnitude larger than the *K*_q_ reported in ref (43). In order to obtain the correct growth and charge transfer peak at small concentrations, a consequence is that the decrease in charge predicted by eq 14 for *c* > 10^–4^ M is much faster than that observed experimentally. This could potentially be caused by a weaker quenching than that predicted by γ_p_ = 1/(1 + *K*_qp_*c*). For example, fits of the type by γ_p_ = 1/(1 + *K*_qp_*c*)^α^, with α ≈ 1/3, give substantially better fits for concentrations above 1 mM, but these functions lack physical justification within the model used here and will therefore not be pursued further. We will see in the next section that another model allows better fits at large concentrations with a value more comparable to that reported in ref (43).

In the case of zero added salt *c* = 0, one obtains
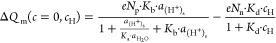
15

**Figure 4:**
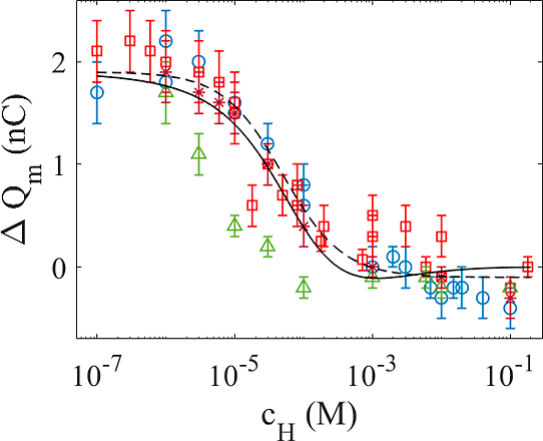
Measured contact charge transfer vs concentration of acid for HCl (blue circles), H_2_SO_4_ (red squares), HNO_3_ (green triangles), and HCOOH (brown stars). The dashed line is a fit of eq 15 to the data with *N*_p_ = 4.0 × 10^10^, , *K*_d_ = 2 × 10^4^ M^–1^, and *N*_n_ = 1.2 × 10^10^, whereas the solid line is a fit of eq 18 to the data with *Q*_0p_ = 1.9 × 10^–9^ C, *AB*_n_ = 5.1 × •10^–8^ V m^2^, *K*_qn_ = 1 × 10^4^ M^–1^, and *x*_sn_ = 2 × 10^–9^ m.

The dashed line in Figure 4 is a fit to the experimental data with *N*_p_ = 4.0 × 10^10^, , *K*_d_ = 2 × 10^4^, and *N*_n_ = 1.2 × 10^10^. In the particular case of the FEP surface used here, the values for *N*_p_ and *N*_n_ suggest that there are about three times as many sites promoting negative as positive charge on the polymer surface, which is of comparable order of magnitude as reported in ref (43). It is also found that *K*_c_ = 10 K_d_, which may be interpreted to indicate that the interaction between hydrogen ions and polymer sites forming negative charge is considerably stronger than the corresponding interactions at the positive sites.

As opposed to Figure 3, there is no peak observed in Figure 4. The reason for this is that the bulk hydrogen ions move in to occupy sites contributing to positive charge opposing the already existing negative charge associated with surface proton activity mediated by water or the charge due to additional cations, and they continue to do so until saturation as given in eq 11. Therefore, adding acids results in a decrease and even reversal of charge transfer, as observed in Figure 4. It should be pointed out that the theory presented here does not account for any quenching of activity due to high concentrations of bulk hydrogen ions. No such quenching effects are found within the concentration range investigated here, but it could be present at higher concentrations. It should also be pointed out that it is possible that there is a correlation between the surface and bulk activity of hydrogen ions as in eq 2. Comparing eq 15 with the available experimental data, one does not find any evidence of such a correlation to within the uncertainty of the measurements. Thus, one could conclude that there might not be such a correlation or that it cannot be detected with the experimental uncertainty of the technique used. In addition, there is also a possibility that the design of the experiment and the used concentration range do not allow detection of such correlations, but extending the experiment to smaller or larger concentrations would lead to additional challenges that are outside the scope of the current work.

### 3.3 Removal of Ions in the Diffuse Part of the Electrical Double Layer

A shortcoming of the theory in the previous section is that it is based on chemical equilibrium constants which are difficult to relate directly to contact electrification. Specific and untested assumptions about the behavior of sites such as , , and  are made to explain that charge can be removed and contribute to charge transfer. This allows one to estimate the sign of the removed charge and also when it crosses over from positive to negative. However, it does not explain how much charge is removed or from which spatially charged region it is removed from.

A remedy for this latter problem can be found using an extended version of the theory presented in ref (41). Here, it is assumed that the flow of the water front removes positive charge in the diffuse electrical double layer. In ref (41), the origin of these sites was not clarified. However, we can do that by leaning on the theory presented in Section 3.2. Even in the absence of any added salt, the surface activity of hydrogen ions is not zero and will contribute to charge transfer due to sites like . According to eq 13, one has , and the charge transfer in deionized water is explained as a combination of surface proton activity and the ease at which ions are released through the equilibrium constant *K*_b_. Here, we will assume that this charge *Q*_0p_ is released into water when the three-phase contact line is passing by. This can be understood if one assumes that the charge released is determined by the organization of the hydrogen bond network (e.g., asymmetries and topological defects) near the surface since the moving contact line will disrupt this network. On the other hand, the addition of external ions (either *c* > 0 or *c*_H_ > 0 in the experiments) is of a different origin and forms a part of the electrical double layer in the normal sense. Therefore, when the contact line passes through the region of concentrated charge depicted in Figure 1, there must be a plane of shear distance *x*_s_ from the hydrophobic surface beyond which the externally introduced ions will move away due to the shear force when the water front moves past it. Experiments done suggest that complete charge transfer may occur whether the contact line is moved relatively slowly or more quickly, and the experiments are, therefore, expected to provide a shear force larger than the minimum shear force required to remove the charge using the three-phase contact line. In each situation, the plane of shear should, therefore, be located at a fixed distance *x*_sp_.

To find the charge contribution from the externally introduced ions, we start by considering the situation where the bulk hydrogen ion activity is zero such that *c*_H_ = 0. To get an estimate, we assume as in ref (41) that the potential as a function of the position *x* (in nanometers) in the electrical double layer can be expressed as φ = *B* exp(−κ*x*), where *B* is constant (typically *B* ∼ −0.1 V) and the inverse Debye length is given by  (nm^–1^) at room temperature.^47^ The area over which the charge is removed from the fluid is *A* = *wL*, where w is the horizontal width of the electrode and L is the effective extension near the metal electrode edge where charge is collected. Near sites such as , the Stern layer is negatively charged such that *B* = *B*_p_ < 0, and positive counterions in the diffuse layer give rise to removal of a positive charge Δ*Q*_dp_ > 0. Upon withdrawal of the polymer surface from water, the positive charge in the diffuse layer is removed beyond a distance *x*_sp_, and a net negative charge remains on the polymer surface. This net negative polymer surface charge induces a net positive charge in the underlying metal electrode. The net positive charge Δ*Q*_dp_ removed from the electrical double layer due to the passing contact line is found by integrating the charge density from *x*_sp_ to infinity as done in ref (41), i.e.,
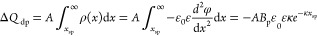
16where ε_0_ is the permittivity of vacuum and ε = 80 is the relative permittivity of water. It is known that the permittivity decreases as one approaches the solid surface as the ions are dehydrated, but such details will not be considered in the model given here. We will assume that the net positive charge Δ*Q*_dp_ removed from the electrical double layer is given by eq 16, which was introduced in ref (41). The total charge may, therefore, be expected to be the sum of *Q*_0p_ and Δ*Q*_dp_. However, this is only valid in the absence of quenching. At finite ion concentrations, the activity is quenched according to γ_p_ = 1/(1 + *K*_qp_*c*) as introduced in ref (43) and discussed in Section 3.1. Note that this quenching factor applies to all the charge available in the electrical double layer for charge transfer. The charge transfer induced in the metal electrodes can, therefore, be written as
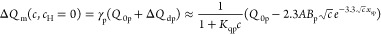
17

The solid line of Figure 3 shows a fit of eq 17 to the experimental data with *Q*_0p_ = 1.7 × 10^–9^ C, *AB*_p_ = −5.4 × 10^–7^ V m^2^, *K*_qp_ = 20 M^–1^, and *x*_sp_ = 60 × 10^–9^ m. Since *w* = 1.0 × 10^–2^ m, one gets *L* = *A*/*w* = 5 × 10^–4^ m for the vertical length if *B*_p_ = −0.1 V. This could be interpreted as charge is collected over about half a millimeter in the vicinity of the metal electrode edge. The value *K*_qp_ = 20 × M^–1^ for the equilibrium quenching constant found here is orders of magnitude smaller than the value *K*_qp_ = 1.0 × 10^4^ M^–1^ found in the previous section. However, it should also be pointed out that the value *K*_qp_ = 20 M^–1^ is of the same order of magnitude as the values of the corresponding constant quenching equilibrium constant *K*_q_ found in figure 5 in ref (43). Moreover, the fit of eq 17 to the experimental data is much better than that obtained using eq 14 at higher concentrations, which could suggest that a slower falloff and a smaller quenching equilibrium constant may have merits. However, it should also be mentioned that uncertainty in the experimental data is too large to confirm or reject the plateau in the charge vs concentration seen using eq 17 at concentrations between 1 and 10 mM.

For sites such as , the Stern layer is positively charged due to the contributions from the bulk hydrogen ion concentration *c*_H_, such that *B* = *B*_n_ > 0, and negative counterions in the diffuse layer give rise to a charge Δ*Q*_n_ < 0. Upon withdrawal of the polymer surface from water, the negative charge in the diffuse layer is removed beyond a distance *x*_sn_, and a net positive charge remains on the polymer surface. This net positive polymer surface charge induces a net negative charge in the underlying metal electrode.

Using similar arguments as when deriving eq 17, taking into account these to contributions and assuming that they are independent, the charge induced in the metal electrode is now
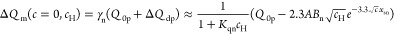
18

In eq 18, a quenching factor of the type γ_n_ = 1/(1 + *K*_qn_*c*_H_), where *K*_qn_ is an equilibrium constant, has been invoked to better fit the experimental data. Its main function is to reduce the peak of the negative term in eq 18, which otherwise would be significant. The solid line of Figure 4 shows a fit of eq 18 to the experimental data with *Q*_0p_ = 1.9 × 10^–9^ C, *AB*_n_ = 5.1 × 10^–8^ V m^2^, *K*_qn_ = 1 × 10^4^ M^–1^, and *x*_sn_ = 2 × 10^–9^ m.

The fit of eq 18 to the experimental data suggests that *x*_sn_ is only 2 nm, while *x*_sp_ obtained by fitting eq 17 to the experimental data is 60 nm. It may be possible to understand that there is weaker attachment to sites such as *P*_s_^n^ than *P*_s_^p^, such that shear forces more easily remove ions from the hydrogen ion sites. Additionally, one also notes a possible size effect in that the hydrogen ion may take very little space in the electrical double layer. However, these numbers in themselves are only suitable for qualitative comparison, as previous experiments using another polymer surface suggested that *x*_sp_ could be as low as 10 nm.^41^

The model in eq 18 also suggests that the quenching is stronger in the case of hydrogen ions, and the quenching constant *K*_qn_ has the same order of magnitude as the equilibrium constant *K*_d_ in eq 15. It should be pointed out that both eqs 15 and 18 correctly predict the crossover from positive to negative charge. However, eq 18 also suggests that there is a peak and subsequent decrease in negative charge as the concentration increases. Within the range of concentrations investigated in the current work, such a phenomenon could not be determined with confidence given the uncertainty in the measured data.

### 3.4 Outlook

The two theories presented in this work build on refs (41) and (43) and assume that ions are responsible for the charge transfer. However, they also differ in some fundamental aspects. For example, the theory presented in ref (43) features an acid-base equilibrium at the solid–liquid interface, with exchange of OH^–^ and/or H^+^ between the polymer and water. However, the origin of the negative charge associated with the interface is highly debated,^6−10^ and a recent model suggests that the explicit presence of hydroxide ions is not needed for a negative charge to develop at a water surface.^11^ Herein, we take that view, which means that, for example, the species  are not due to free hydroxide ions adsorbing to sites on the polymer surface but rather due to asymmetries and defects that allow the formation of negative sites near the polymer surface. This assumption brings an asymmetry to the way one treats hydrogen ions and hydroxide ions and is different from the theory of ref (43).

The new part introduced in Section 3.2 is an intermediate step where the surface hydrogen ions contribute to the electrical double layer through eqs 1–3. Here, they compete with other cations (eq 4), and the surface charge density depends on the fraction of hydrogen ions and cations contributing to the electrical double layer (eq 7). As in ref (43), it is assumed that the water activity is quenched as the ion concentration increases. Unlike ref (43), the chemical equilibrium theory of Section 3.2 states that anions (B−) do not give rise to a significant contribution, which is in line with the experimental data reported in the current work. For example, adding NaOH and NaCl to the solution is seen to give rise to very similar charge transfer behavior as seen in Figure 4. It may be that at higher concentrations of hydroxide ions, the surface proton activity is altered, which one may see indications in Figure 4, but this is not investigated further here.

As for the theory presented in Section 3.3, it combines the theories in refs (41) and (43) and Section 3.2 in an attempt to extract more detail about the electrical double layer contribution to the charge transfer. The theory provided in Section 3.3 provides a better fit to the experimental data with quenching equilibrium constants comparable to that of the acid–base equilibrium theory of ref (43). If correct, it is, therefore, likely that also other contact electrification experiments as those in refs (39) and (41) can be explained by the same type of charge mechanism.

One unresolved question remains why the acids investigated do not all appear to show zero charge transfer at the same concentration. This appears to occur at about 0.1 mM for HNO_3_, near 1 mM for HCl at variable concentrations, and near 10 mM or higher or sometimes not at all for H_2_SO_4_. These variations could of course just be due to the fluctuations and inherent uncertainties in the technique used, but given the large variability that claim may appear improbable. One may speculate whether the charge transfer reversal variations are either due to the hydrogen ions or specific anion effects. While HCl, HCOOH, and HNO_3_ all may release one hydrogen ion, H_2_SO_4_ may release two per molecule. The sulfate and nitrate ions have roughly comparable hydration shell diameters. Based on these facts, one may imagine that the addition of H_2_SO_4_ may require more water molecule coordination and a corresponding reduced water activity at the surface, which leads to slower falloff in charge transfer with concentration. If this mechanism is correct, nitrate ions should, therefore, be associated with the largest water activity near the polymer surface since adding HNO_3_ appears to exhibit reversal of charge transfer at lower concentrations than the other acids. However, more research studies on the coordination and surface-specific properties of ions are needed to obtain better insight, but this is outside the scope of the current work.

One may be tempted to question the ion-based charge transfer theory investigated in the current work given that it is well known that contact between solids and insulating oils also gives rise to significant charge transfer.^50,51^ In fact, this has been a significant problem, for example, in the transport of insulating liquids in pipes.^50,51^ Research has shown that water may help stabilize static charges, but it is not needed for charge transfer.^52^ The presence of contact electrification in the absence of mobile ions in some nonaqueous liquids has been claimed to be evidence that electrons are responsible.^39,53^ However, given the available experimental data, such a conclusion appears premature. As pointed out in Section 3.2, the surface activity of ions may explain the charge transfer that occurs in pure deionized water. The observation that the charge transfer at small ion concentrations does not change much is seen in both Figure 3 and Figure 4. This may appear to support the theory of eqs 14 and 17 at small concentrations, although one cannot from this make a conclusive statement that ions alone are responsible for charge transfer in deionized water. One may also speculate whether the surface activity of ions or charged groups at the interface of a solid contacting an oil may be responsible for charge transfer. Upon contact, shear may result in release of ions or rearrangement of charge in the surface groups at the solid surface. As an example, shear forces during impact may rearrange the surface groups and reveal new charge configurations in a temperature-dependent manner. Charge transfer timescale, polarity in different liquids, and microscopic surface charge arrangement need to be studied in situ in more detail before a conclusion of origin of the charge transfer species can be reached.

The details of the charge transfer upon contact between water and an inert solid are also very complex since water molecules are highly polarizable and may contribute to the charge transfer in several different manners.^9−11^ In this work, models based entirely on charge transfer due to ions were presented. The surface activity of protons was used to explain the charge transfer that occurs in pure water, and additional ions resulted in addition charge transfer. It was not ruled out that electrons could transfer when the inner electrical double layer first forms or when the hydrogen network is disrupted, but these processes do not contribute to the additional charge transfer observed when ions are added to the solution.

The model in Section 3.2 did not state the spatial region from which the released charge came from, although it was presumed to come from an electrical double layer. The model presented in Section 3.3 assumed that externally introduced charges participate in the diffuse part of the double layer and contribute to charge transfer. This assumption is similar to the assumptions made in electrokinetic models used to model the zeta potential,^10,35,47^ but it should be noted that in the current situation, the charge transfer occurs during the passage of the three-phase contact line over the hydrophobic polymer near the location of the metal electrode edge. Ultimately, the shear force required to remove the ions—while the opposite polarity remains behind—may depend on the detailed flow pattern and the dynamic contact angle in the microscale region near the hydrophobic surface. The water flow pattern for an advancing contact angle larger than 90^◦^ is probably associated with streamlines bending downward (when the polymer film is moving down) or upward (when the polymer film is moving up) without any split injection or ejection.^54^

The movement of the three-phase contact line to facilitate charge transfer is undertaken in a range of different recent studies.^33,38−43^ In refs (38)–^41^, there are clear indications of the same type of charge transfer as observed in the current work for chlorides, such that a peaked charge transfers at a given ion concentration. Although in most studies the peak charge transfer occurs in the range 0.01–1 mM,^38−41^ some studies suggest a peak at higher concentrations closer to 0.1 M.^33^ However, yet other studies have not reported such peak behavior at all, see, e.g., ref (42) and references therein. In many cases, the surface can be engineered to obtain an ion-specific response.^16,19,34^ Clearly, the surface properties and the attraction and leaching of ions play an important role through factors such as the equilibrium constants and the hydrogen ion surface activity in eq 13. For example, setting  instead of 0.38 in eq 14 gives rise to the dotted curve in Figure 3. This type of curve does not exhibit a peak and does at least qualitatively resemble the behavior observed in ref (42). Thus, it is likely that the specific surface and the corresponding surface hydrogen activity may have a significant impact on the charge transfer at small ion concentrations and should therefore warrant further investigation. If the solid surface is engineered to accommodate specific ions, then the theory in Section 3 would need revision to be applicable.

In a recent study, it was demonstrated that zwitterions do not influence the electrostatic forces in the electrical double layer.^55^ The influence of neutral species on the contact electrification may further reveal whether distance from the solid surface plays a role. The model presented in the previous section only accounts for the distance between the polymer surface and the removed ions through a lumped parameter (*x*_sn_ or *x*_sp_), and further experiments with neutral spacers may reveal whether these also influence the ion removal.

## 4 Conclusions

In this work, the contact electrification upon dipping an inert, hydrophobic solid surface into water was investigated. In order to measure the charge transfer, a metal electrode was covered by the hydrophobic surface such that it did not come in contact with the aqueous solution. It was found that a wide range of aqueous solutions exhibited a similar behavior, in which the charge transfer first increased and subsequently decreased with ion concentration. For acids, the charge transfer decreased monotonously and even reversed at sufficiently high concentrations. From these observations, it seems likely that the ion specificity does not play a particular role, with exception of hydrogen ions. In order to explain the results, two different models based on ion transfer from the electrical double layer are discussed and show to explain the experimental data reasonably well.
